# GANT61, a GLI inhibitor, sensitizes glioma cells to the temozolomide treatment

**DOI:** 10.1186/s13046-016-0463-3

**Published:** 2016-11-28

**Authors:** Jianlong Li, Jinquan Cai, Shihong Zhao, Kun Yao, Ying Sun, Yongli Li, Lingchao Chen, Ruiyan Li, Xiuwei Zhai, Junhe Zhang, Chuanlu Jiang

**Affiliations:** 1 Department of Neurosurgery, The Second Affiliated Hospital of Harbin Medical University, 246 Xuefu Road, Nangang 150086 Harbin, People’s Republic of China; 2 Neuroscience Institute, Heilongjiang Academy of Medical Sciences, Harbin, 150086 China; 3 Department of Pathology, Sanbo Brain Hospital, Capital Medical University, Beijing, 100093 China; 4 Department of Neurosurgery, Huashan Hospital, Fudan University, Shanghai, 200040 China; 5 Chinese Glioma Cooperative Group (CGCG), Beijing, 100050 China; 6 Department of Neurosurgery, Daqing LongNan Hospital, Daqing, 163001 China

**Keywords:** Glioma, GANT61, Temozolomide, Hedgehog, DNA damage, O^6^-methylguanine DNA methyltransferase (MGMT), Notch1 pathway

## Abstract

**Background:**

The aim of this study was to investigate the effect of downregulating Hedgehog pathway by GANT61 on human glioma cells, examine the consequent changes of temozolomide (TMZ)-induced effects and explore the molecular mechanisms.

**Methods:**

The cytotoxicity of a Gli1/2 inhibitor, GANT61 was examined both alone and in combination with TMZ in human glioma cell lines. The mRNA and protein expression alterations were determined by quantitative real-time polymerase chain reaction (qRT-PCR) and Western blot, respectively. CCK-8 assay detected the cell proliferative capability. Apoptotic cell number was measured by flow cytometry. The transwell assay was used to test the cell invasive capability. DNA damage effect was identified by COMET assay and γH2AX expression.

**Results:**

Proliferation of tumor cells treated with GANT61 in combination with TMZ was significantly suppressed compared with those treated with either drug used alone. The combination treatment induced a higher rate of apoptosis, DNA damage and reduced the invasive capability of glioma cells. DNA damage repair enzyme MGMT and the Notch1 pathway increased in the cells treated by TMZ treatment. However, GANT61 could abrogated the protein increasing.

**Conclusions:**

GANT61 sensitizes glioma cells to TMZ treatment by enhancing DNA damage effect, decreasing MGMT expression and the Notch1 pathway.

**Electronic supplementary material:**

The online version of this article (doi:10.1186/s13046-016-0463-3) contains supplementary material, which is available to authorized users.

## Background

Glioma is the most common primary malignant brain tumor in adults [1, 2]. Despite the development of aggressive comprehensive treatments, the median overall survival (OS) of patients with glioblastoma (GBM), the most malignant type of glioma, has only marginally changed over the past 25 years and still remains about 12 to 15 months [3–6]. Thus, novel therapeutic approaches to cure this malignancy are urgently needed.

Temozolomide (TMZ) is an oral chemotherapeutic drug for GBM which penetrates into the brain and damages DNA by the induction of DNA O^6^-methylguanine [4, 7–10]. However, the DNA repair enzyme O^6^-methylguanine methyltransferase (MGMT) could abrogate the cytotoxic O^6^-methylguanine DNA adduct before it causes harm, which may be the important mechanism of TMZ resistance [11, 12]. Indeed, lower response to TMZ treatment has been observed in patients with high MGMT and inactivation of the MGMT gene is associated with longer patient survival [13, 14].

In addition, the Hedgehog (Hh) pathway involving in development and cancer, is indispensable for epithelial-to-mesenchymal transition (EMT), metastasis and invasion, cancer stemness, and chemoresistance [15–17]. Moreover, the inhibition of the activity of GLI, the functional transcription activators of the Hh pathway, can interfere with almost all DNA repair types in human cancer, which indicates an important role of Hh/Gli in tumor cells’ survival from DNA damage induced by chemotherapy or radiotherapy [18]. Sonic hedgehog could promote tumor proliferation in a Notch-dependent manner [19–22]. The Hh and Notch pathways interact to control the EMT/MET [19]. Hes-1, a primary target gene of Notch1, can be activated by both Notch and Hh signaling [23–25].

Thus, Hh signaling may serve as an important therapeutic target to overcome DNA repair induced by TMZ treatment and consequently, to increase the chemotherapeutic response in the treatment of cancer. In the present study, we investigated the effect of Gli1/2 downregulation by GANT61 on TMZ induced cytotoxicity in U87 and U251 glioma cells. By comparing the effects of GANT61, TMZ treatment, and their combination in glioma cell proliferation, invasion/EMT, and apoptosis, we found a synergistic action between GANT61 and TMZ treatment. Our results revealed the important role of GLI inhibitor GANT61 in enhancing the sensitivity of glioma cell for TMZ.

## Methods

### Datasets used in this study

Gene expression profiling datasets were obtained from the Chinese Glioma Genome Atlas (CGGA) [26] (http://www.cgga.org.cn), including the mRNA array of 305 samples and RNA sequencing of 105 samples.

### Tumor cell line

Human GBM cell lines U251 and U87 were purchased from the Chinese Academy of Sciences Cell Bank. A human oligodendroglia cell line (OL) was a kind gift from Prof. Fengmin Zhang of Harbin Medical University. The cells were maintained in a 37 °C, 5% CO2 incubator in Dulbecco’s Modified Eagle’s Medium (DMEM, Corning, USA) supplemented with 10% fetal bovine serum (FBS, Biological Industries, Israel). We assembled the Cancer Cell Line Encyclopedia (CCLE) (www.broadinstitute.org/ccle) and screened 208 differential expressive genes (Additional file 1A, Fold changes ≥ 2), including GFAP, S100B, IGFBP2 (Additional file 1B) and so on.

### Reagents

TMZ (Sigma-Aldrich, USA) and GANT61 (Selleck Chemical, USA) were dissolved in 10% dimethylsulfoxide (DMSO, Sigma) to create a stock solution (TMZ: 100 mg/ml; GANT61: 10 mM). DMSO concentration was kept below 0.1% in all the cell cultures and did not exert any detectable effect on cell growth or cell death. Recombinant Human Shh (rShh, 1 μg/mL) was purchased from R&D, Minnesota, USA. Primary antibodies used for Western Blot analysis were rabbit anti-Gli1 (CST, USA), Gli2 (Proteintech, USA), N-cadherin (CST), Vimentin (Abcam, England), Fibronectin (Abcam), Notch1 (Abcam), Hes1 (Abcam), MGMT (Proteintech), γH2AX (CST), GAPDH (Wanleibio, China) and mouse anti-E-cadherin (CST); horseradish peroxidase–conjugated secondary antibody (goat anti-rabbit or anti-mouse) were purchased from ZSGB-BIO.

### Cell viability assay

The cytotoxicity of TMZ and GANT61 on U87 and U251 cells was determined by Cell Counting Kit-8 (CCK-8, Dojindo, Japan) assay. Tumor cells were seeded at 2-5 × 10^3^ cells/well (0.1 ml) in 96-well flat bottom plates and incubated overnight at 37 °C. After exposure to the above described treatment for 24, 48, and 72 h, CCK-8 (10 μl, 10%) was added to each well once every hour before incubation ended. Then its absorbance at 450 nm was measured by a microplate reader (IMARK). All experiments were repeated in triplicate.

### Apoptosis detection

U251 and U87 cells were plated in 6-well plates and treated with TMZ or GANT61 or a combination of the two. The apoptosis ratio was analyzed 48 h post-treatment *via* using Annexin V-FITC Apoptosis Detection Kit (BD Biosciences, San Diego, CA) according to the manufacturer’s instructions. Annexin V-FITC and propidium iodide (PI) double staining was used to evaluate the percentages of apoptosis. Annexin V^−^ and PI^−^ cells were used as controls. Annexin V^+^ and PI^−^ cells were designated as apoptotic, and Annexin V^+^ and PI^+^ cells as necrotic. Tests were repeated in triplicate.

### In vitro invasion assays

Transwell membranes coated with Matrigel (BD Biosciences, San Jose, CA) were used to assay the invasive ability of glioma cells in vitro. Treated cells were plated at 5 × 10^4^ per well in the upper chamber in serum-free medium. FBS 10% was added to the medium in the lower chamber. After incubation for 24 h, non-invading cells were removed from the top well with a cotton swab, while the bottom cells were fixed with 95% ethanol, stained with 0.1% crystal violet, and photographed in three independent 10× fields for each well. Three independent experiments were conducted and used to calculate fold migration relative to the blank control while the error was calculated as the standard error (SE).

### Western blot

Cell lysates were harvested, equivalent amounts of total protein were separated by 10% SDS polyacrylamide gel electrophoresis (SDS-PAGE), and transferred to polyvinylidene difluoride (PVDF) membranes. After blocking with 5% fat-free milk and 0.1% Tween-20 in tri-buffered saline with Tween (TBST) for 1.5 h at room temperature, membranes were incubated with diluted anti-Gli1, Gli2, E-cadherin, N-cadherin, Vimentin, Fibronectin, MGMT, Notch1, Hes1, γH2AX (Ser139) and anti-GAPDH primary antibodies. Horseradish peroxidase-conjugated anti-mouse or anti-rabbit secondary antibodies were used, and bound antibodies were detected using the ECL system.

### Quantitative RT-PCR (qRT-PCR) analysis

Total RNA was extracted using Trizol Reagent (Invitrogen, USA) according to the manufacturer’s instructions. Total cDNA was reversely transcribed from 1 μg of total RNA (Perfect Real Time, Takara, Japan). To quantify gene expression, two-step qRT-PCR was performed using a FastStart Universal SYBR Green Master (ROX) by Roche LightCycler^R^ Real Time System. Expression levels were normalized to glyceraldehyde-3-phosphate dehydrogenase (GAPDH). The PCR conditions were hot-start at 95 °C (15 s), with annealing and extension at 60 °C (60 s) for 40 cycles, followed by a melting curve analysis. All qRT-PCR data were analyzed using the 2^-ΔΔCt^ method. And the primers used are shown in Additional file 2.

### COMET assay

The comet assay (Trevigen, Gaithersburg, MD) was performed according to the manufacturer’s protocol using alkaline conditions. Cell samples were handled under dimmed light to prevent DNA damage from ultraviolet light. Combine cells at 1 × 10^5^/ml with molten LMAgarose and immediately pipette 50 μl onto CometSlide. After placing slides flat at 4 °C for 10 min, immerse slides in lysis solution for 60 min and freshly prepared Alkaline Unwinding Solution, pH > 13 for 20 min. Electrophoresis was carried out at the rate of 1.0 V/cm for 30 min. The slides were removed from the electrophoresis chamber, washed in deionized water for 5 min and in ice cold 70% ethanol for 5 min. Subsequently, the slides were air-dried, and DNA was stained with 50 μl of SYBR Green I dye (Sangon Biotech, 1:10,000 in Tris-EDTA buffer, pH 7.5) for 30 min and immediately analyzed using a fluorescence microscope (Axiovert 200, Carl Zeiss). Data was analyzed using CometScore (TriTek, Sumerduck, VA).

### Statistical analysis

Gene set variation analysis (GSVA) with Gli expression was analyzed by GSVA package [27] of R (https://www.r-project.org/). Data are presented as mean ± SD. All statistical analyses were performed using SPSS version 13.0 software (Chicago, IL, USA). The Student’s *t*-test was used to analyze the difference between the means of treatment and control groups. One-way analysis of variance (ANOVA) was used to analyze the significance among three or more groups, and the Fisher’s least significant difference (LSD) method was used for multiple comparisons when the probability for ANOVA was statistically significant. Data were considered statistically significant at *P* <0.05 level.

## Results

### Gli1 is a prognostic marker in glioma and participates in variety of biological behaviors

**Fig. 1:**
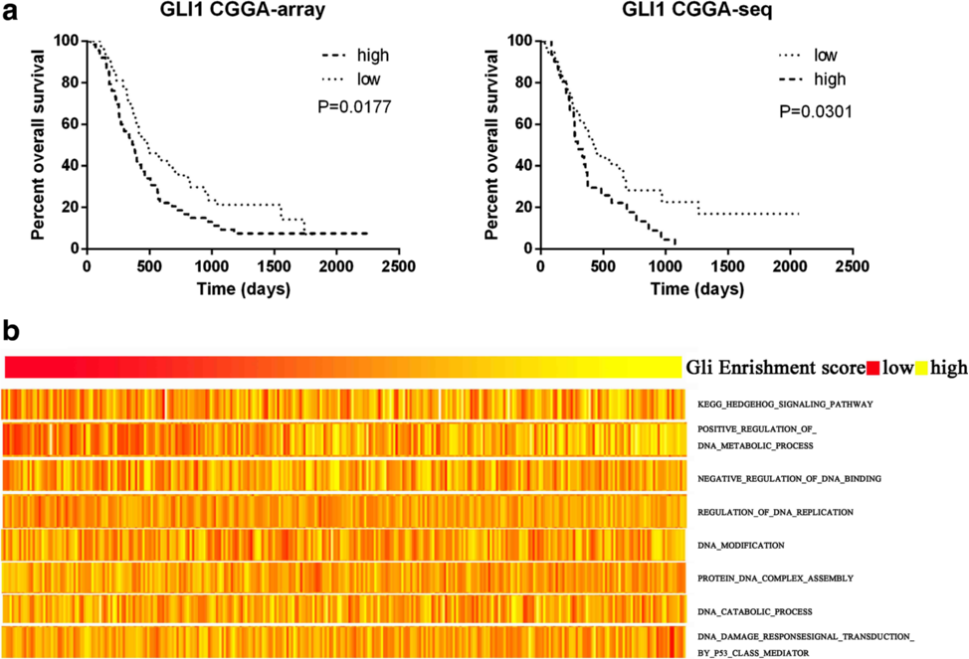
Gli1 is a prognostic marker in glioma and participates in variety of biological behaviors. **a** A Kaplan-Meier test estimates the survival for glioma patients. Patients with low expression of Gli1 had a favorable prognosis in the CGCG-array and CGGA-seq (*P* = 0.0177 and *P* = 0.0301, respectively). **b** GSVA with GLI expression. Gene expression signatures of glioma-associated biological behaviors, including the “Hh signaling pathway”, “DNA metabolic process (positive regulation)”, “DNA binding (negative regulation)”, “DNA replication”, “DNA modification”, “protein-DNA complex assembly”, “DNA catabolic process” and “DNA damage response signal transduction by the p53 class mediator” were generated from the Molecular Signature Database. The GLI expression went higher from left to right. A high enrichment score indicates positive correlation with GLI expression and a low enrichment score indicates the reverse

As shown in Fig. 1a, patients with glioblastoma containing high or low expression of Gli1 showed a considerably different prognosis in the CGGA mRNA array dataset and RNA sequencing dataset. Patients with low expression of Gli1 had a favorable prognosis in CGCG (*P* = 0.0177 and *P* = 0.0301, respectively). Cox regression analysis also revealed that GLI expression was associated with the overall survival of patients with glioblastoma from the CGGA mRNA and RNA sequencing datasets (*P* = 0.019, HR = 1.588, 95% CI = 1.080–2.334; *P* = 0.032, HR = 1.687, 95% CI = 1.045–2.725, respectively).

Gene set variation analysis (GSVA) illustrated that GLI was associated with several biological behaviors besides the Hh pathway, including positive regulation of the DNA metabolic process and DNA damage response signal transduction, and negative regulation of DNA binding and protein-DNA complex assembly (Fig. 1b). Up- and downregulated genes associated with indicated biological behaviors went positively and negatively related to GLI expression. These function analyses were related to DNA metabolism, replication, DNA damage repair and glioma malignant progression. Therefore, we thought to find out the role of Hh inhibitor GANT61 in glioma cells’ survival.

### Reduction in the survival of human glioma cells after treatment with GANT61

**Fig. 2:**
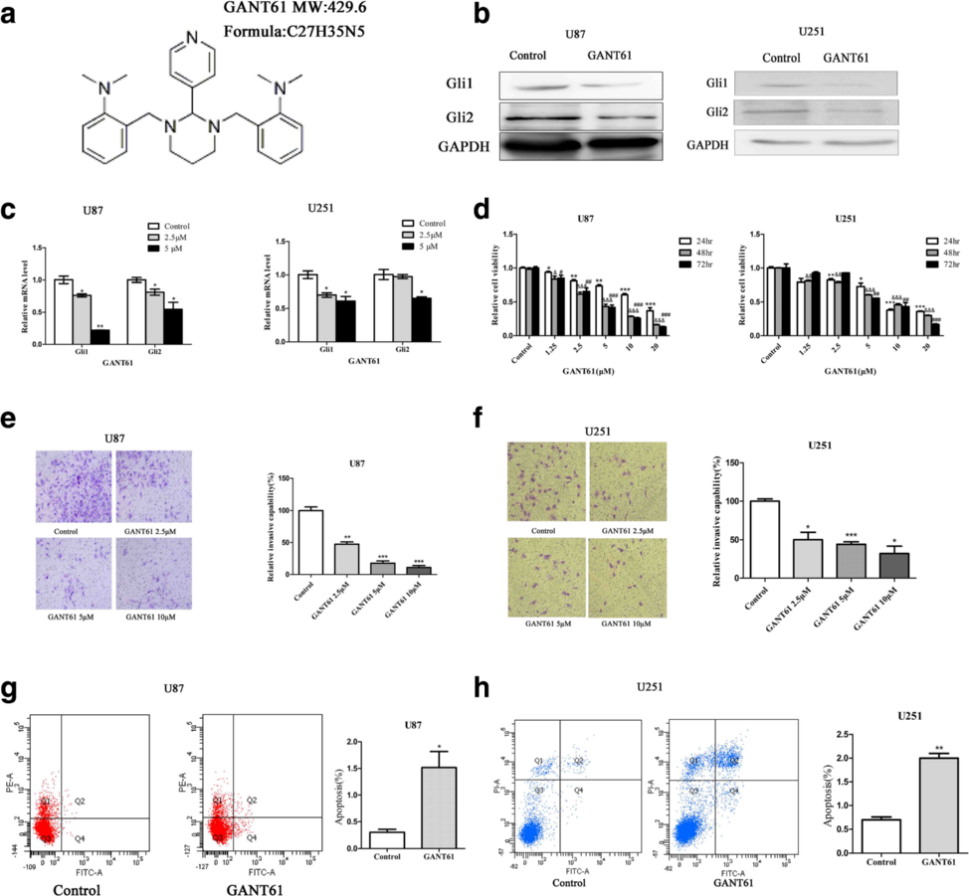
GANT61 inhibited the survival of human glioma cells in vitro. **a** Chemical structure and molecular weight of GANT61. **b** U87 and U251 glioma cells were treated with GANT61 (5 μM) for 48 h. Gli1 and Gli2 expression were determined by Western blot and (**c**) qRT-PCR analysis. GAPDH was used as the loading control. **d** U87 and U251 cells were cultured in 96-well plates, respectively. Relative proliferation of glioma cells treated with different doses of GANT61 for different durations were determined by CCK-8. GANT61 treatment significantly decreased cells proliferation in a dose- and time-dependent manner. **e**, **f** GANT61 inhibited invasion. Transwell assay showed that the invasive capability of U87 or U251 cells treated with GANT61 (5 μM) was weaker than the control in a dose-dependent manner. **g**, **h** GANT61 induced apoptosis. U87 and U251 cells were either solvent-treated (Control) or challenged with GANT61 for 36 h, and then, cell apoptosis was examined by flow cytometry assay using the Annexin V/PI. Annexin V analysis showed that glioma cells treated with GANT61 displayed greater apoptosis than the control group. Data are from one of three representative experiments. The error bars represent standard error. Data are shown as mean ± SEM for the three replicates. Statistical significance levels are indicated as *P < 0.05, **P < 0.01, ***P < 0.001 compared with control group

In our present study, we confirmed that GANT61 (Fig. 2a) could inhibit the Gli1/2 expression (Fig. 2b and c. *P < 0.05, **P < 0.01). To clarify the action of GANT61 as a single antitumor agent, we first performed a CCK-8 assay using U87 and U251 glioma cells treated with GANT61 at concentrations of 1.25 to 20 μM for 24, 48, and 72 h, respectively. As shown in Fig. 2d, GANT61 exerted an inhibitory effect on glioma cells in a dose and time dependent manner (*, &, # *P* < 0.05; **, && and ## *P* < 0.01; ***, &&& and ### *P* < 0.001 compared to Control). The concentration of inhibited cell viability (IC_50_) was 10.24 μM for 24 h, 4.38 μM for 48 h, and 4.39 μM for 72 h in U87 cells, while it was 7.38 μM for 48 h, and 7.59 μM for 72 h in U251 cells. However, treatment with GANT61 at the same concentration for the same amount of time did not induce significant cytotoxicity of human OL cells, except for those treated at 20 μM (Additional file 3). The transwell assay revealed that the numbers of invasive cells at different concentrations of GANT61 were significantly reduced compared with the control group (Fig. 2e and f. * *P* < 0.05, **P < 0.01, ***P < 0.001). Next, we observed that the population of apoptotic cells was larger among the glioma cells treated with GANT61 than the untreated cells (Fig. 2g and h. *P < 0.05, **P < 0.01).

### GANT61 enhances the suppression of TMZ for the glioma cells’ biological behavior

**Fig. 3:**
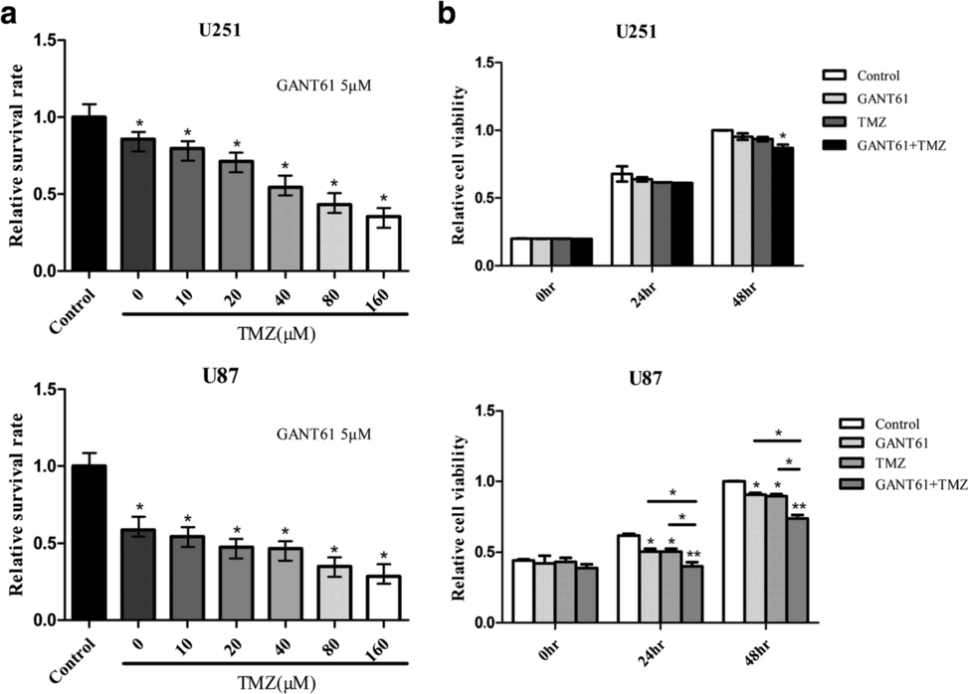
GANT61 enhanced the proliferation-inhibiting effect of TMZ. **a** Cells were cultured in 96-well plates and then treated with solvent only (Control) or increasing doses of TMZ with 5 μM GANT61 for 48 h. Relative proliferation of U87 and U251 cells treated with GANT61 and different doses of TMZ were determined by CCK-8. **b** Cells were seeded into 96-well plates and then treated with Control, GANT61 (1 μM), TMZ (60 μM) and GANT61 + TMZ for 24 and 48 h. Cell viability was examined using the CCK-8 assay. GANT61 + TMZ treatment resulted in a significant decrease in cell proliferation. Data are mean ± SEM for the three replicates. *P < 0.05, **P < 0.01

To detect the cytotoxicity of combining TMZ and GANT61, we first used a CCK-8 assay to determine the sensitivity of U251 to the TMZ. The result showed that the medial IC_50_ was 124 μM and 99 μM at 48 h and 72 h, respectively (Additional file 4). We referred to previous reports [28, 29] for the TMZ sensitivity of U87. As shown in Fig. 3b, the combined effect of TMZ and GANT61 exhibited the highest of proliferation inhibition effect (*P < 0.05, **P < 0.01 vs. control at 24 h and 48 h). This GANT61–induced potentiation of the effects of TMZ was recognized at a lower concentration than that needed to induce cytotoxicity with TMZ alone. Isobologram analyses indicated that the effects of the combinations of GANT61 and TMZ in both U87 and U251 were both synergistic.

**Fig. 4:**
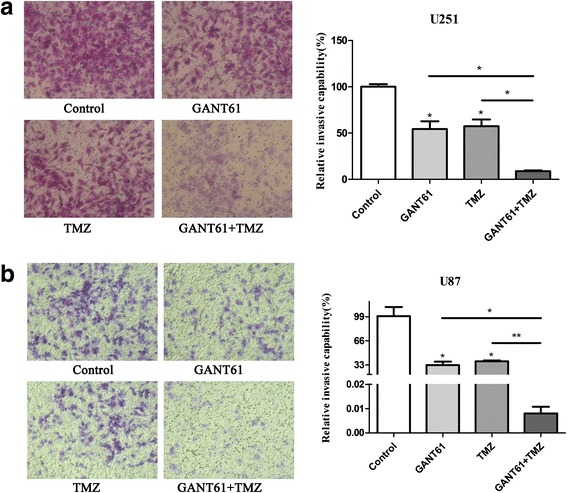
GANT61 increased TMZ-induced invasion in glioma cells. Cell invasion was analyzed using the Transwell assay. Transwell assay showed the invasive capability of (**a**) U251 and (**b**) U87 cells treated with GANT61, TMZ and the combination therapy. GANT61 or TMZ reduced the invasion of glioma cells and their combination showed much lower invasion as demonstrated by representative microscope graphs (10 × 30). *P < 0.05, **P < 0.01 compared with control group. The results were presented as the mean of three independent experiments

Then, a transwell assay was performed to demonstrate that TMZ plus GANT61 suppressed the invasive capability of glioma cells. As shown in Figs. 2e, f and 4, the invaded cell numbers in the GANT61 group were smaller than those in the Control group, showing a negative effect of GANT61 on the invasion ability of U251 and U87 cells (*P* < 0.05). Compared to the TMZ treatment, the combination of GANT61 and TMZ treatment caused a more significant reduction of the invasion rate (Fig. 4. *P < 0.05, **P < 0.01).

**Fig. 5:**
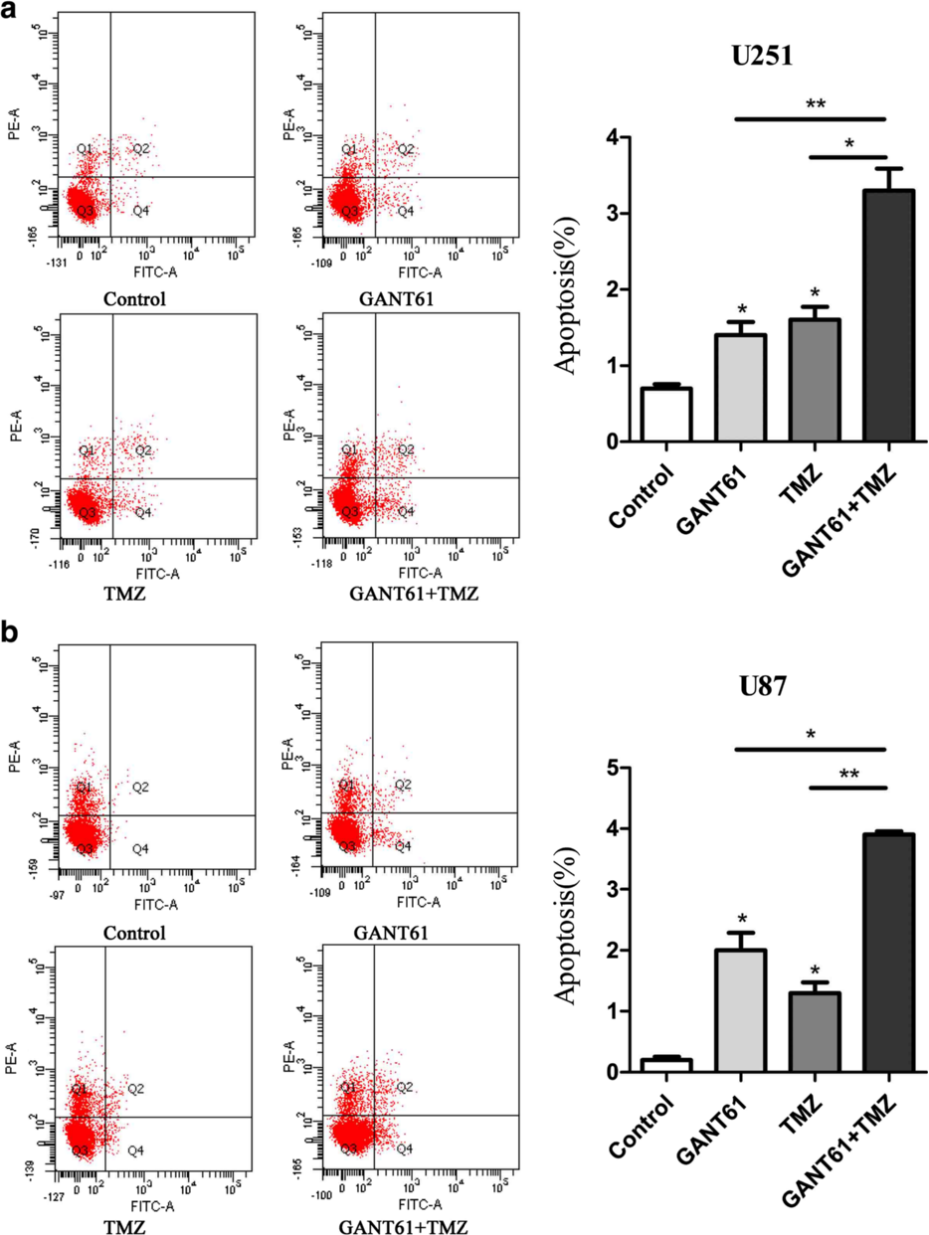
GANT61 increased apoptosis of glioma cells treated with TMZ. Apoptosis of cells under different treatments were detected by flow cytometric analysis with Annexin V-FITC/PI staining. **a** U251 and (**b**) U87. *Left*: The representative quadrantal diagrams showing results of flow cytometric analysis. *Right*: Histogram manifested average apoptosis rates in cell groups of different treatments. GANT61 and TMZ treatment resulted in only a slight increase in apoptosis. *P < 0.05. Combined GANT61 and TMZ treatment resulted in a significant increase in early apoptosis rate compared to cells treated with GANT61 or TMZ alone, *P < 0.05, **P < 0.01. All data were representative of three independent experiments

Next, after 2 days of treatment with the combination of TMZ and GANT61, a significant increase in the number of apoptotic cells was noted when compared to TMZ or GANT61 used alone at the same dose (Fig. 5. *P < 0.05, **P < 0.01). The result indicated that GANT61 enhances TMZ-induced apoptosis.

### Treatment with the combination of TMZ and GANT61 reverses epithelial-to-mesenchymal transition (EMT) of glioma cells

**Fig. 6:**
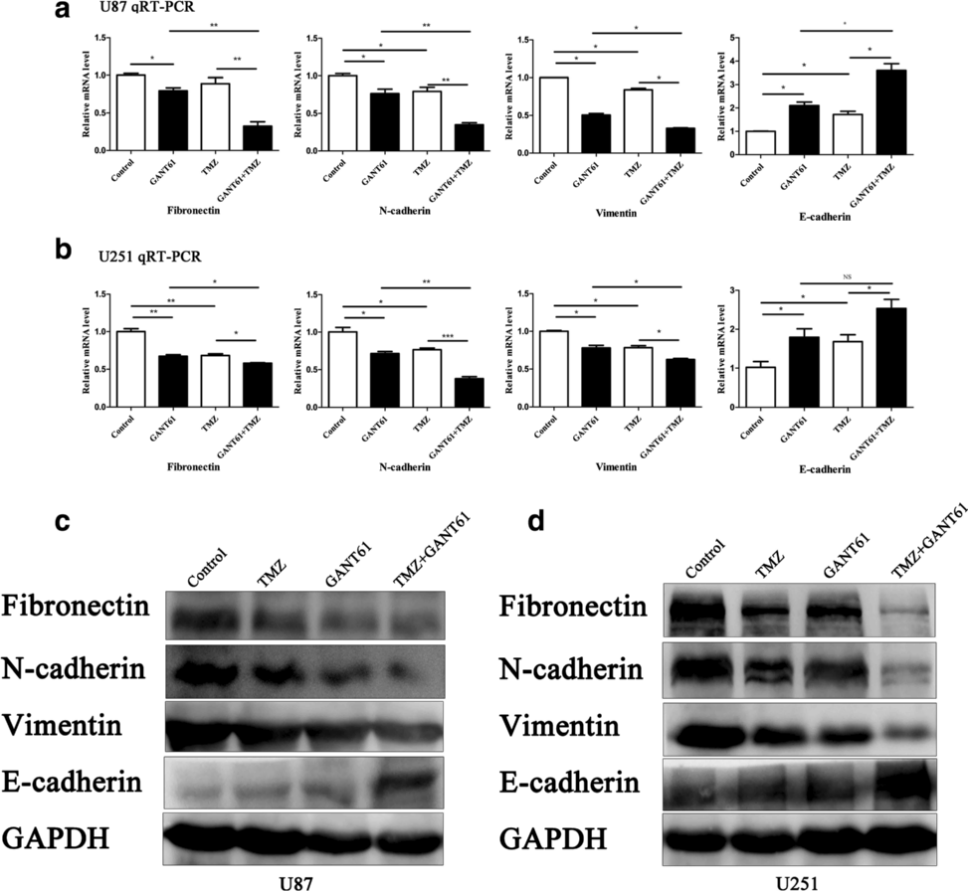
Combining GANT61 and TMZ treatment reversed EMT of glioma cells. **a** The mRNA expression levels of different genes. qRT-PCR analysis demonstrated that either GANT61 or TMZ treatment resulted in a reduction of N-cadherin, Vimentin and Fibronectin mRNA expression and an elevation of E-cadherin mRNA expression. The combination of GANT61 and TMZ treatment led to more significant decreases in N-cadherin, Vimentin and Fibronectin mRNA expression and increase in E-cadherin mRNA expression than cells treated with GANT61 or TMZ treatment alone. *P < 0.05, **P < 0.01, ***P < 0.001. **b** Western blot analysis showed that the protein expression of N-cadherin, Vimentin and Fibronectin decreased, and the protein expression of E-cadherin increased in cells, especially in the GANT61 + TMZ group, while only a slight change was detected in the TMZ group. This experiment was repeated three times

QRT-PCR analysis revealed a significant increase of epithelial cadherin (E-cadherin) mRNA expression and a significant decrease of mesenchymal markers N-cadherin, Vimentin, and Fibronectin expression in GANT61 treated glioma cells (Fig. 6a and b). Interestingly, glioma cells also exhibited lower transcript levels of N-cadherin, Vimentin and Fibronectin, and upregulated E-cadherin transcript after incubation with 100 μM TMZ for 48 h. Compared with TMZ treatment alone, combined TMZ and GANT61 induced a further reduction of N-cadherin, Vimentin, and Fibronectin expression and augmented the elevation of E-cadherin transcript (Fig. 6a and b). Further, we tested whether these treatments could modulate the expression of proteins that regulate EMT. Results of Western blot were manifested according to the protein level alterations. Similar conclusions could be drawn based on Western blot analysis. (Fig. 6c and d).

### GANT61 enhances DNA damage effect initiated by TMZ treatment

**Fig. 7:**
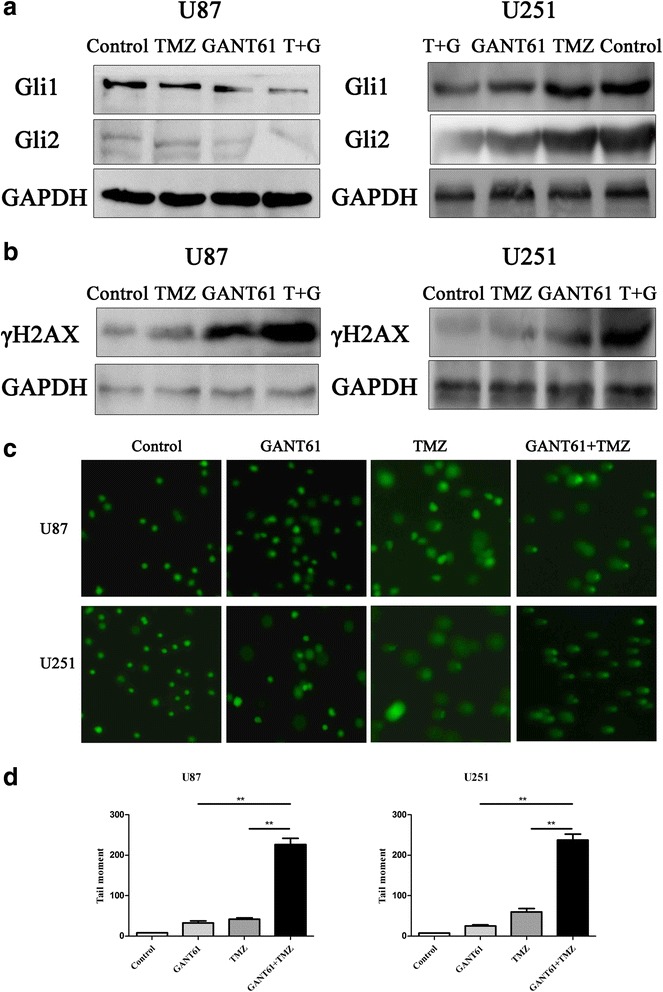
GANT61 enhanced the DNA damage effect initiated by TMZ treatment. **a** Western blot analysis showed an obviously decreased expression of Gli1 and Gli2 in cells treated with a combination of GANT61 and TMZ, compared to TMZ-treated U251 and U87 glioma cells. Cells were continuously exposed to 5 μM GANT61 or 100 μM TMZ for up to 48 h. GAPDH was used as the loading control. **b** U87 and U251 cells were treated with 5 μM GANT61, 100 μM TMZ or a combination of the two treatments for 48 h. The γH2AX levels were measured by immunoblot assay. **c** Representative comet staining images in U87 and U251 cells after different treatments (5 μM GANT61, 100 μM TMZ or both). **d** Quantitative assessment of comet tail moment. **P < 0.01

To elucidate the mechanisms by which the combination of TMZ and GANT61 lead to more tumor cells growth retardation or apoptosis, glioma cells were treated with GANT61 (5 μM), TMZ (100 μM) or both of them for 48 h. Gli1 and Gli2, which are associated with TMZ resistance and TMZ-activated DNA damage repair, were obviously down-regulated compared with the single therapy groups (Fig. 7a).

Phosphorylated histone H2AX (γH2AX) is used as a biomarker of cellular response to DNA double-strand breaks (DSBs), and its potential for monitoring DNA damage and repair in human populations has been explored in many studies [30, 31]. Herein, the expression of γH2AX was examined following exposure to GANT61 and (or) TMZ for up to 40 h. TMZ treatment alone triggered phosphorylation of H2AX slightly, whereas a much higher level of γH2AX was observed after GANT61 was added (Fig. 7b), suggesting substantially more unresolved DNA DSBs in the combination treatment versus single-treated cells.

In addition, single cells were analyzed by the COMET assay, which detects DNA damage by altering the pattern of cellular elution through agarose gels. Compared to groups treated with TMZ or GANT61 alone, significant changes in elution profiles were detected in the combination of TMZ and GANT61-treated cells by fluorescence microscopy, Tail Moment and Tail Length (Fig. 7c and d). This indicated a role of DNA damage signaling in combination of TMZ and GANT61-induced cytotoxicity. Collectively, data demonstrated that the combination of Hh-inhibitor and TMZ result in more evidently reduced cell growth, increased cell death and induction of DNA damage that is associated with reduced Gli1 and Gli2 protein levels. These findings emphasize the significance of the Hh signaling pathway in human glioma cell growth and survival, regulated at the level of the GLI genes.

### GANT61 abrogates the MGMT, Notch1, and Hes1 overexpression induced by TMZ treatment

**Fig. 8:**
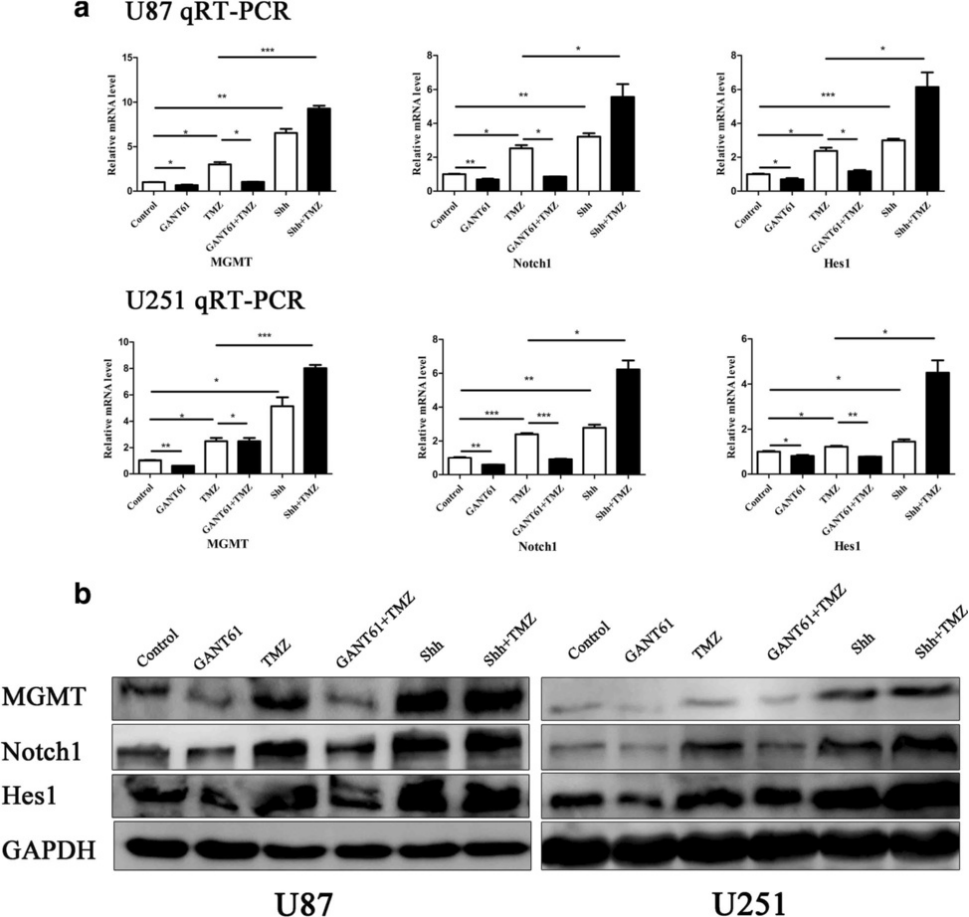
GANT61 abrogated the MGMT, Notch1, and Hes1 overexpression induced by TMZ treatment. **a** qRT-PCR analysis of MGMT, Notch1, and Hes1 mRNA expression after incubation with GANT61, TMZ, GANT61 + TMZ, Shh, and Shh + TMZ for 48 h. Under TMZ, Shh or Shh + TMZ treatment, MGMT, Notch1, and Hes1 mRNA levels were much higher than the indicated treatment (TMZ vs. Control; Shh vs. Control; Shh + TMZ vs. TMZ). GANT61-treated cells showed decreased MGMT, Notch1, and Hes1 mRNA expression, especially in GANT61 + TMZ (GANT61 vs. Control; GANT61 + TMZ vs. TMZ). *P < 0.05, **P < 0.01, ***P < 0.001. **b** Western blot analysis revealed increased MGMT, Notch1, and Hes1 protein expression in U87 and U251 glioma cells treated with TMZ. However, the GANT61 + TMZ group had lower levels of MGMT, Notch1, and Hes1 protein than the TMZ group when the same dose of TMZ was used, while GANT61 showed a lower expression than the control group and Shh + TMZ group had higher expression than the TMZ group. The results shown were representative of three independent experiments

Glioma cells treated with TMZ exhibited significant increases in MGMT, Notch1 and Hes1 mRNA, and protein levels (Fig. 8a and b). However, these upregulations were suppressed by GANT61, as shown in Fig. 8. Also, MGMT, Notch1 and Hes1 expression levels in the GANT61 group were obviously lower than those in the control group (*P* < 0.05, **P < 0.01). To further demonstrate the importance of the Hh pathway in the regulation of MGMT and the Notch1 pathways, treatments of Shh, or a combination of Shh and TMZ were performed, and the results showed increased MGMT, Notch1, and Hes1 expression following Shh exposure. Moreover, Shh augmented the TMZ-induced effect when combined with Shh and TMZ, compared to TMZ alone. (*P < 0.05, ***P < 0.001). This indicated a regulatory role of GANT61 in the regulation of expression of MGMT and the Notch1 pathway. However, further study is needed to confirm this.

## Discussion

GBM, characterized by a high proliferative and invasive capability, is refractory to traditional surgical tumor resection supplemented with chemotherapy or radiotherapy. Thus, the outcome of GBM patients remains poor [32]. Alkylating agents, including TMZ and carmustine, readily cross the blood–brain barrier and show cytotoxicity against GBM [11]. TMZ induces DNA lesion O^6^-methylguanine, and this would activate the repair pathways in feedback, accounting for TMZ-resistance [33]. Emerging literatures have revealed that Hh signaling affects almost all DNA repair types by enabling tumor cells to survive DNA damage induced by chemotherapy [18]. In addition, activating mutations in the Hh pathway causes a large percentage of tumors [34]. GLIs are the effector molecules of the Hh pathway and therefore we investigated the role of GLI-specific inhibitor GANT61 in glioma survival. In our study, GANT61 alone could dampen the proliferation of glioma cells. Moreover, GANT61 potentiates TMZ cytotoxicity due to inhibiting DNA damage-repair initiated by TMZ treatment. Treatment with the same doses (2.5 to 10 μM) for the same period (24/48 h) does not cause significant damage to OL cells, which suggests appropriate doses for studying *in vivo* or clinical investigation. This is conducive to the development of precision treatment.

Simultaneously, elevated expression of the repair protein MGMT also results in TMZ resistance [33]. MGMT can eliminate the cytotoxic O6-methylguanine DNA adduct [11]. In our study, we found that TMZ treatment increased the expression of MGMT, but is abolished by GANT61. This suggests that blocking the Hh pathway enhances the cytotoxicity of TMZ in glioma cells by downregulating the expression of MGMT. As described above, GANT61 sensitized glioma cells to TMZ *via* enhancing apoptosis and inhibiting proliferation/invasion. However, the downstream factors mediating these effects remain to be investigated. The expression of a series of tumor suppressors and oncogenes relating to cancer cell apoptosis, invasion and TMZ resistance were compared among glioma cells treated with TMZ or GANT61 or a combination of the two. We found that Notch1 and Hes1 were significantly upregulated in TMZ-treated glioma cells, but not in the combination of TMZ and GANT61-treated glioma cells. Notch1 and Hes1 have been demonstrated to be important oncogenes playing critical roles in glioma cell survival and TMZ resistance [35]. Furthermore, the Notch pathway interacts closely with the Hh pathway. For instance, Notch directly suppresses Hh *via* Hes1-mediated inhibition of Gli1 transcription [36]. Ectopic Notch activation increases Gli2 protein [37]. Hh signaling promotes proliferation of monociliated choroid plexus tumor cells in a Notch-dependent pathway [21]. Inactivating Hh results in decreased expression of the Hh/Notch signaling component [20]. Inhibiting the Hh pathway suppresses Notch signaling and induces a mesenchymal-to-epithelial-like transition [19]. Both the Hh and Notch pathways participate in tumorigenesis and TMZ resistance. Thus, it is logical to suppose that glioma cells resistant to TMZ and protect themselves partly through the activity of Notch1 pathway, while GANT61 somehow abrogated this upregulation, reducing the chemoresistance and at last sensitizing glioma cells to TMZ. The activation of the Notch pathway after TMZ therapy was suspected to confer DNA damage repair. However, further study is required to confirm this.

EMT is a process whereby cells acquire morphologic and molecular alterations that facilitate tumor metastasis and progression. Emerging evidence associates chemoresistance with the acquisition of EMT in cancer [38, 39]. The induced EMT was associated with increased cell motility, invasiveness, and clonogenicity. Hh signaling promotes EMT, and antagonizing Hh signaling inhibits EMT in a GLI-dependent manner [40–44]. Herein, we investigate the combination effect of GLI inhibitor and TMZ on EMT. To the best of our knowledge, this is the first report that treatment with a combination of TMZ and GANT61 can reverse the EMT of glioma cells.

## Conclusion

To conclude, downregulation of the Hh pathway by GANT61 enhances the cytotoxicity of TMZ *via* inhibiting proliferation, suppressing invasion/EMT, and inducing apoptosis. The synergistic mechanism is suggested that down-regulation of the Hh pathway by GANT61, which enhances DNA damage effect and suppresses MGMT and the Notch1 pathway, induces sensitivity to TMZ. These results indicated that GANT61 may represent a potential targeted inhibitor for glioma treatment and may increase the chemo-sensitivity of glioma to TMZ.

## Additional files

Additional file 1: The differential expressive genes between U87 and U251 glioma cell lines. (A) Heatmap showed the differential expressive genes (fold change ≥ 2) between U87 and U251 glioma cell lines. (B) U251 cell line had higher level of GFAP, S100B, and IGFBP2 expression, associated with tumor progression. (JPG 194 kb)
Additional file 2: Gene-specific primers for qRT-PCR analysis. (DOCX 14 kb)
Additional file 3: GANT61 did not appreciably affect the vitality of OL cells at concentrations below 20 μM. NS: not significant. ****P* < 0.001. (JPG 95 kb)
Additional file 4: U251 cells treated with increasing concentrations of TMZ were analyzed using a CCK-8 assay. Statistical significance levels are indicated as: *, ^&^ *P* < 0.05; ***P* < 0.01 and ***, ^&&&^ *P* < 0.001. (JPG 766 kb)

## Abbreviations

CCK-8: Cell counting kit-8
CCLE: Cancer cell line encyclopedia
CGGA: Chinese glioma genome atlas
DMSO: Dimethylsulfoxide
EMT: Epithelial-to-mesenchymal transition
GSVA: Gene set variation analysis
Hh: Hedgehog
MGMT: O^6^-methylguanine DNA methyltransferase gene
OL: Oligodendroglia cell line
OS: Overall survival
PVDF: Polyvinylidene difluoride
qRT-PCR: Quantitative reverse transcriptase PCR
rShh: Recombinant Human Shh
SDS-PAGE: SDS polyacrylamide gel electrophoresis
TMZ: Temozolomide

## References

[1] Cai J, Zhang W, Yang P, Wang Y, Li M, Zhang C, Wang Z, Hu H, Liu Y, Li Q (2015). Identification of a 6-cytokine prognostic signature in patients with primary glioblastoma harboring M2 microglia/macrophage phenotype relevance. PLoS One.

[2] Cai J, Zhu P, Zhang C, Li Q, Wang Z, Li G, Wang G, Yang P, Li J, Han B (2016). Detection of ATRX and IDH1-R132H immunohistochemistry in the progression of 211 paired gliomas. Oncotarget.

[3] Butowski NA, Sneed PK, Chang SM (2006). Diagnosis and treatment of recurrent high-grade astrocytoma. J Clin Oncol.

[4] Stupp R, Mason WP, van den Bent MJ, Weller M, Fisher B, Taphoorn MJ, Belanger K, Brandes AA, Marosi C, Bogdahn U (2005). Radiotherapy plus concomitant and adjuvant temozolomide for glioblastoma. N Engl J Med.

[5] Van Meir EG, Hadjipanayis CG, Norden AD, Shu HK, Wen PY, Olson JJ (2010). Exciting new advances in neuro-oncology: the avenue to a cure for malignant glioma. CA Cancer J Clin.

[6] Liu C-Y, Li Q-J, Cai J-Q (2016). Evolving molecular genetics of glioblastoma. Chin Med J.

[7] Chamberlain MC (2010). Temozolomide: therapeutic limitations in the treatment of adult high-grade gliomas. Expert Rev Neurother.

[8] Annovazzi L, Caldera V, Mellai M, Riganti C, Battaglia L, Chirio D, Melcarne A, Schiffer D (2015). The DNA damage/repair cascade in glioblastoma cell lines after chemotherapeutic agent treatment. Int J Oncol.

[9] Roos WP, Batista LF, Naumann SC, Wick W, Weller M, Menck CF, Kaina B (2007). Apoptosis in malignant glioma cells triggered by the temozolomide-induced DNA lesion O6-methylguanine. Oncogene.

[10] Jiang T, Mao Y, Ma W, Mao Q, You Y, Yang X, Jiang C, Kang C, Li X, Chen L (2016). CGCG clinical practice guidelines for the management of adult diffuse gliomas. Cancer Lett.

[11] Sarkaria JN, Kitange GJ, James CD, Plummer R, Calvert H, Weller M, Wick W (2008). Mechanisms of chemoresistance to alkylating agents in malignant glioma. Clin Cancer Res.

[12] Cai J, Chen J, Zhang W, Yang P, Zhang C, Li M, Yao K, Wang H, Li Q, Jiang C, Jiang T (2015). Loss of ATRX, associated with DNA methylation pattern of chromosome end, impacted biological behaviors of astrocytic tumors. Oncotarget.

[13] Hegi ME, Diserens AC, Godard S, Dietrich PY, Regli L, Ostermann S, Otten P, Van Melle G, de Tribolet N, Stupp R (2004). Clinical trial substantiates the predictive value of O-6-methylguanine-DNA methyltransferase promoter methylation in glioblastoma patients treated with temozolomide. Clin Cancer Res.

[14] Qiu ZK, Shen D, Chen YS, Yang QY, Guo CC, Feng BH, Chen ZP (2014). Enhanced MGMT expression contributes to temozolomide resistance in glioma stem-like cells. Chin J Cancer.

[15] Shahi MH, Rey JA, Castresana JS (2012). The sonic hedgehog-GLI1 signaling pathway in brain tumor development. Expert Opin Ther Targets.

[16] Varnat F, Duquet A, Malerba M, Zbinden M, Mas C, Gervaz P, Ruiz i Altaba A (2009). Human colon cancer epithelial cells harbour active HEDGEHOG-GLI signalling that is essential for tumour growth, recurrence, metastasis and stem cell survival and expansion. EMBO Mol Med.

[17] Syn WK, Jung Y, Omenetti A, Abdelmalek M, Guy CD, Yang L, Wang J, Witek RP, Fearing CM, Pereira TA (2009). Hedgehog-mediated epithelial-to-mesenchymal transition and fibrogenic repair in nonalcoholic fatty liver disease. Gastroenterology.

[18] Meng E, Hanna A, Samant RS, Shevde LA (2015). The impact of hedgehog signaling pathway on DNA repair mechanisms in human cancer. Cancers (Basel).

[19] Xie G, Karaca G, Swiderska-Syn M, Michelotti GA, Kruger L, Chen Y, Premont RT, Choi SS, Diehl AM (2013). Cross-talk between Notch and Hedgehog regulates hepatic stellate cell fate in mice. Hepatology.

[20] Doyle AJ, Redmond EM, Gillespie DL, Knight PA, Cullen JP, Cahill PA, Morrow DJ (2015). Differential expression of Hedgehog/Notch and transforming growth factor-beta in human abdominal aortic aneurysms. J Vasc Surg.

[21] Li L, Grausam KB, Wang J, Lun MP, Ohli J, Lidov HG, Calicchio ML, Zeng E, Salisbury JL, Wechsler-Reya RJ (2016). Sonic Hedgehog promotes proliferation of Notch-dependent monociliated choroid plexus tumour cells. Nat Cell Biol.

[22] Bertrand FE, Angus CW, Partis WJ, Sigounas G (2012). Developmental pathways in colon cancer: crosstalk between WNT, BMP, Hedgehog and Notch. Cell Cycle.

[23] Wall DS, Mears AJ, McNeill B, Mazerolle C, Thurig S, Wang Y, Kageyama R, Wallace VA (2009). Progenitor cell proliferation in the retina is dependent on Notch-independent Sonic hedgehog/Hes1 activity. J Cell Biol.

[24] Sang L, Roberts JM, Coller HA (2010). Hijacking HES1: how tumors co-opt the anti-differentiation strategies of quiescent cells. Trends Mol Med.

[25] Wall DS, Wallace VA (2009). Hedgehog to Hes1: the heist of a Notch target. Cell Cycle.

[26] Cai J, Yang P, Zhang C, Zhang W, Liu Y, Bao Z, Liu X, Du W, Wang H, Jiang T, Jiang C (2014). ATRX mRNA expression combined with IDH1/2 mutational status and Ki-67 expression refines the molecular classification of astrocytic tumors: evidence from the whole transcriptome sequencing of 169 samples samples. Oncotarget.

[27] Hanzelmann S, Castelo R, Guinney J (2013). GSVA: gene set variation analysis for microarray and RNA-seq data. BMC Bioinf.

[28] Kanzawa T, Germano IM, Kondo Y, Ito H, Kyo S, Kondo S (2003). Inhibition of telomerase activity in malignant glioma cells correlates with their sensitivity to temozolomide. Br J Cancer.

[29] Kanzawa T, Germano IM, Komata T, Ito H, Kondo Y, Kondo S (2004). Role of autophagy in temozolomide-induced cytotoxicity for malignant glioma cells. Cell Death Differ.

[30] Huang X, Halicka HD, Darzynkiewicz Z: Detection of histone H2AX phosphorylation on Ser-139 as an indicator of DNA damage (DNA double-strand breaks). Curr Protoc Cytom 2004, Chapter 7:Unit 7 27. 10.1002/0471142956.cy0727s30 18770804

[31] Gupta SK, Kizilbash SH, Carlson BL, Mladek AC, Boakye-Agyeman F, Bakken KK, Pokorny JL, Schroeder MA, Decker PA, Cen L, et al. Delineation of MGMT Hypermethylation as a Biomarker for Veliparib-Mediated Temozolomide-Sensitizing Therapy of Glioblastoma. J Natl Cancer Inst 2016, 108. 10.1093/jnci/djv369 PMC4862419 26615020

[32] Tobias A, Ahmed A, Moon KS, Lesniak MS (2013). The art of gene therapy for glioma: a review of the challenging road to the bedside. J Neurol Neurosurg Psychiatr.

[33] Goellner EM, Grimme B, Brown AR, Lin YC, Wang XH, Sugrue KF, Mitchell L, Trivedi RN, Tang JB, Sobol RW (2011). Overcoming temozolomide resistance in glioblastoma via dual inhibition of NAD+ biosynthesis and base excision repair. Cancer Res.

[34] Ng JM, Curran T (2011). The Hedgehog’s tale: developing strategies for targeting cancer. Nat Rev Cancer.

[35] Hiddingh L, Tannous BA, Teng J, Tops B, Jeuken J, Hulleman E, Boots-Sprenger SH, Vandertop WP, Noske DP, Kaspers GJ (2014). EFEMP1 induces gamma-secretase/Notch-mediated temozolomide resistance in glioblastoma. Oncotarget.

[36] Schreck KC, Taylor P, Marchionni L, Gopalakrishnan V, Bar EE, Gaiano N, Eberhart CG (2010). The Notch target Hes1 directly modulates Gli1 expression and Hedgehog signaling: a potential mechanism of therapeutic resistance. Clin Cancer Res.

[37] Ringuette R, Atkins M, Lagali PS, Bassett EA, Campbell C, Mazerolle C, Mears AJ, Picketts DJ, Wallace VA (2016). A Notch-Gli2 axis sustains Hedgehog responsiveness of neural progenitors and Muller glia. Dev Biol.

[38] Yan YR, Xie Q, Li F, Zhang Y, Ma JW, Xie SM, Li HY, Zhong XY (2014). Epithelial-to-mesenchymal transition is involved in BCNU resistance in human glioma cells. Neuropathology.

[39] Li H, Da LJ, Fan WD, Long XH, Zhang XQ (2015). Transcription factor glioma-associated oncogene homolog 1 is required for transforming growth factor-beta1-induced epithelial-mesenchymal transition of non-small cell lung cancer cells. Mol Med Rep.

[40] Xu X, Su B, Xie C, Wei S, Zhou Y, Liu H, Dai W, Cheng P, Wang F, Xu X, Guo C (2014). Sonic hedgehog-Gli1 signaling pathway regulates the epithelial mesenchymal transition (EMT) by mediating a new target gene, S100A4, in pancreatic cancer cells. PLoS One.

[41] Bai Y, Lu H, Wu C, Liang Y, Wang S, Lin C, Chen B, Xia P (2014). Resveratrol inhibits epithelial-mesenchymal transition and renal fibrosis by antagonizing the hedgehog signaling pathway. Biochem Pharmacol.

[42] Amantini C, Morelli MB, Nabissi M, Cardinali C, Santoni M, Gismondi A, Santoni G: Capsaicin triggers autophagic cell survival which drives epithelial mesenchymal transition and chemoresistance in bladder cancer cells in an Hedgehog-dependent manner. Oncotarget. 2016. 10.18632/oncotarget.10326 PMC5226576 27367032

[43] Ke Z, Caiping S, Qing Z, Xiaojing W (2015). Sonic hedgehog-Gli1 signals promote epithelial-mesenchymal transition in ovarian cancer by mediating PI3K/AKT pathway. Med Oncol.

[44] Tang C, Mei L, Pan L, Xiong W, Zhu H, Ruan H, Zou C, Tang L, Iguchi T, Wu X (1850). Hedgehog signaling through GLI1 and GLI2 is required for epithelial-mesenchymal transition in human trophoblasts. Biochim Biophys Acta.

