# Supplementary figures and images for: GANT61, a GLI inhibitor, sensitizes glioma cells to the temozolomide treatment
Source: J Exp Clin Cancer Res. 2016 Nov 28;35:184. doi: 10.1186/s13046-016-0463-3 (PMC5127098; doi:10.1186/s13046-016-0463-3)

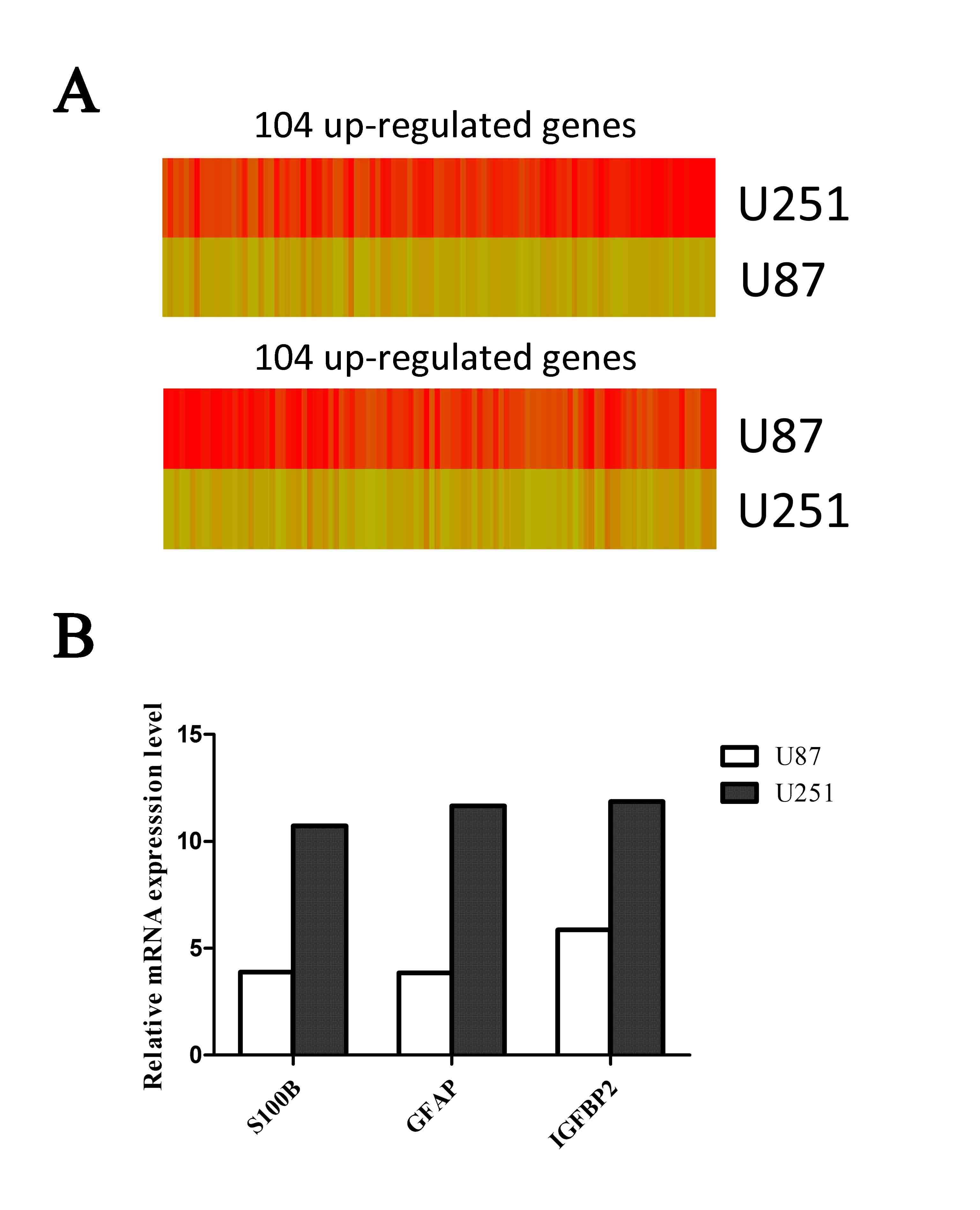

Supplement: Additional file 1: — The differential expressive genes between U87 and U251 glioma cell lines. (A) Heatmap showed the differential expressive genes (fold change ≥ 2) between U87 and U251 glioma cell lines. (B) U251 cell line had higher level of GFAP, S100B, and IGFBP2 expression, associated with tumor progression. (JPG 194 kb) [file 13046_2016_463_MOESM1_ESM.jpg]

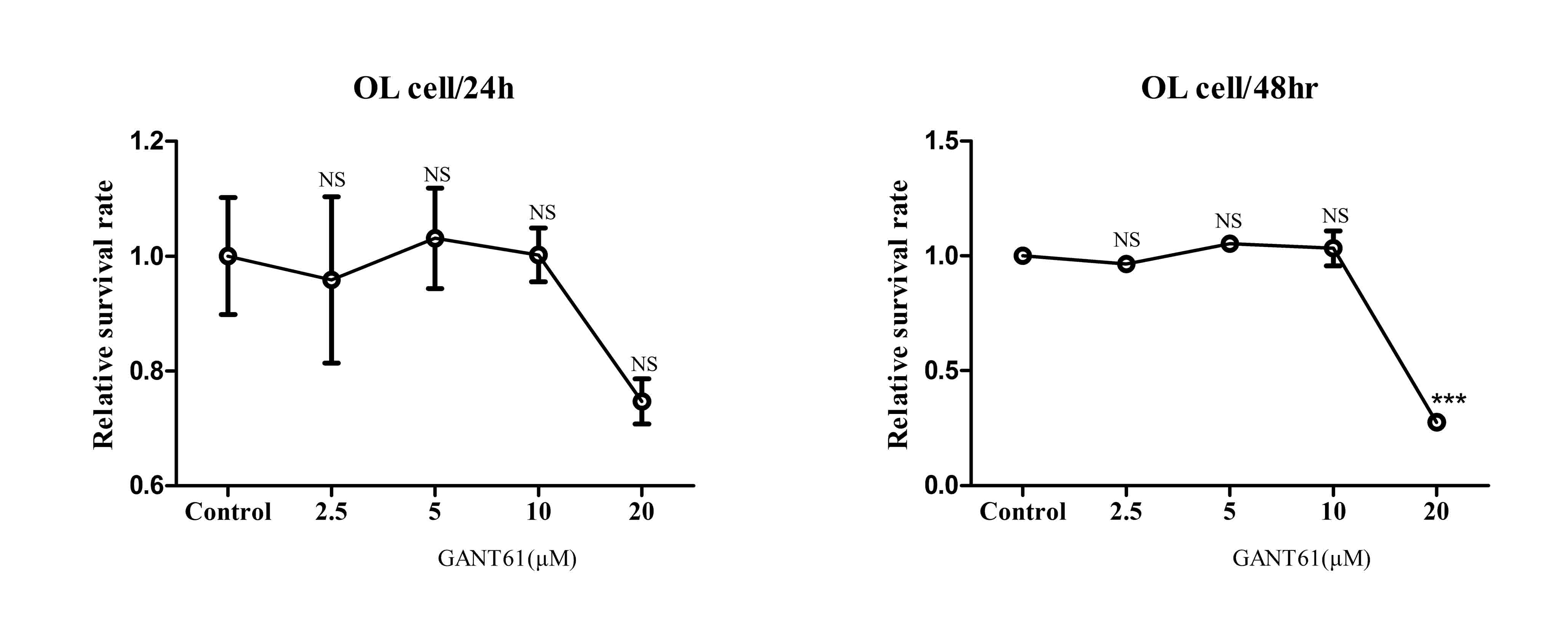

Supplement: Additional file 3: — GANT61 did not appreciably affect the vitality of OL cells at concentrations below 20 μM. NS: not significant. ***P < 0.001. (JPG 95 kb) [file 13046_2016_463_MOESM3_ESM.jpg]

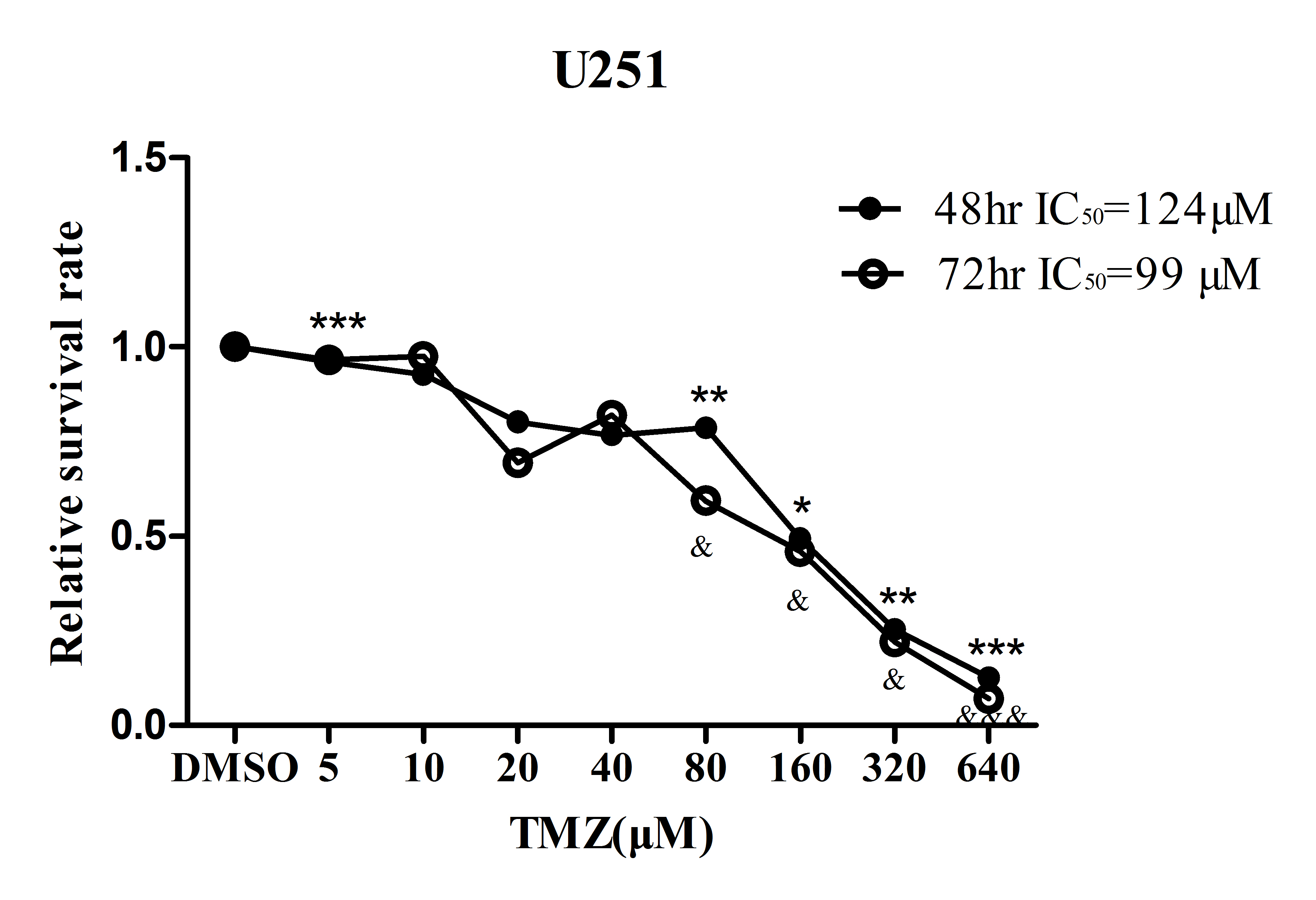

Supplement: Additional file 4: — U251 cells treated with increasing concentrations of TMZ were analyzed using a CCK-8 assay. Statistical significance levels are indicated as: *, & P < 0.05; **P < 0.01 and ***, &&& P < 0.001. (JPG 766 kb) [file 13046_2016_463_MOESM4_ESM.jpg]
