# Supplementary material for: GANT61, a GLI inhibitor, sensitizes glioma cells to the temozolomide treatment
Source: J Exp Clin Cancer Res. 2016 Nov 28;35:184. doi: 10.1186/s13046-016-0463-3 (PMC5127098; doi:10.1186/s13046-016-0463-3)
Supplement: Additional file 2: — Gene-specific primers for qRT-PCR analysis. (DOCX 14 kb) [file 13046_2016_463_MOESM2_ESM.docx]

**Additional file 2**. Gene-specific primers for qRT-PCR analysis

| **Gli1** | **F 5′- ATCCTTACCTCCCAACCTCTGT -3′** | **Fibronectin** | **F 5′-** **ATCACCCTCACCAACCTCAC -3′** |
| --- | --- | --- | --- |
|  | **R 5′-** **AACTTCTGGCTCTTCCTGTAGC -3′** |  | **R 5′-** **TCCCTCGGAACATCAGAAAC -3′** |
| **Gli2** | **F 5′-** **TGTAAGCAGGAGGCTGAGGT -3′** | **MGMT** | **F 5′-** **CCTGGCTGAATGCCTATTTC -3′** |
|  | **R 5′-** **GCTCGTTGTTGATGTGATGC -3′** |  | **R 5′-** **TGTCTGGTGAACGACTCTTGC -3′** |
| **N-cadherin** | **F 5′-** **CTGACAATGACCCCACAGC -3′** | **Notch1** | **F 5′-** **TGAATGGCGGGAAGTGTGAA -3′** |
|  | **R 5′-** **TCCTGCTCACCACCACTACTT -3′** |  | **R 5′-** **ATAGTCTGCCACGCCTCTG -3′** |
| **E-cadherin** | **5′- TGATTCTGCTGCTCTTGCTG -3′** | **Hes1** | **F 5'-** **GGCTAAGGTGTTTGGAGGCT -3′** |
|  | **R 5′-** **CTCTTCTCCGCCTCCTTCTT -3′** |  | **R 5'-** **GGTGGGTTGGGGAGTTTAGG -3′** |
| **Vimentin** | **F 5′-** **GAAGGAGGAAATGGCTCGTC -3′** | **GAPDH** | **F 5′-** **TGGACTCCACGACGTACTCAG -3′** |
|  | **R 5′-** **CTCAGGTTCAGGGAGGAAAAG -3′** |  | **R 5′-** **CGGGAAGCTTGTCATCAATGGAA -3′** |
